# Pregnancy outcomes in women with adenomyosis, undergoing artificial endometrial preparation with and without gonadotropin-releasing hormone agonist pretreatment in frozen embryo transfer cycles: An RCT

**DOI:** 10.18502/ijrm.v21i6.13635

**Published:** 2023-07-24

**Authors:** Marzieh Eslami Moayed, Ashraf Moini, Ladan Kashani, Maryam Farid Mojtahedi, Tawoos Rezaee, Hamed Tabasizadeh, Khadije Maajani, Nazila Yamini

**Affiliations:** ^1^Department of Gynecology and Obstetrics, Arash Women's Hospital, Tehran University of Medical Sciences, Tehran, Iran.; ^2^Department of Endocrinology and Female Infertility, Reproductive Biomedicine Research Center, Royan Institute for Reproductive Biomedicine, ACECR, Tehran, Iran.; ^3^Breast Disease Research Center (BDRC), Tehran University of Medical Sciences, Tehran, Iran.; ^4^Department of Pediatrics, Clinical Research Development Unit, Bahar Hospital, Shahroud University of Medical Sciences, Shahroud, Iran.; ^5^Department of Epidemiology and Biostatistics, School of Public Health, Tehran University of Medical Sciences, Tehran, Iran.; ^6^Department of Embryology, Arash Women's Hospital, Tehran University of Medical Science, Tehran, Iran.

**Keywords:** Adenomyosis, Embryo transfer, Hormone replacement therapy, Gonadotropin-releasing hormone.

## Abstract

**Background:**

Selecting a suitable and preferable method for endometrial preparation in frozen embryo transfer (FET) cycles for women with adenomyosis is still challenging in infertility treatment.

**Objective:**

To compare 2 artificial endometrial preparation regimens with and without gonadotropin-releasing hormone agonist (GnRHa) pretreatment in women with adenomyosis undergoing FET cycles.

**Materials and Methods:**

This randomized clinical trial study was conducted on 140 adenomyosis cases who underwent FET cycles at Arash Women's hospital, Tehran, Iran from May 2020 to March 2021. Participants were randomly allocated into hormonal replacement therapy (HRT) and HRT+GnRHa pretreatment groups (n = 70/each). Endometrial preparation with 2-6 mg daily estradiol was started in the HRT+GnRHa group, taking after down-regulation with the GnRHa. Within the HRT group, the same dose of estradiol was commenced within the early follicular stage. The main (chemical and clinical pregnancy rates) and auxiliary results (twin pregnancy, miscarriage, and live birth rates) were compared between groups.

**Results:**

The demographic characteristics and severity of adenomyosis, endometrial thickness, and pattern at starting progesterone administration were similar in the 2 groups, and triple-line endometrium was found to be the dominant pattern in both groups (p = 0.65). No significant differences were observed in chemical, clinical, and twin pregnancy rates as well as miscarriage and live birth rates between groups (p = 0.71, p = 0.81, p = 0.11, and p = 0.84, respectively). However, the total estrogen dose and duration of estrogen consumption were significantly higher in the pretreatment group (p = 0.001, and p = 0.003).

**Conclusion:**

These results indicated that the hormonal endometrial preparation with estrogen and progestin for FET cycles is as efficacious as a protocol involving preceding pituitary suppression with a GnRHa. Further large randomized clinical studies are required to confirm these findings.

## 1. Introduction

Adenomyosis is a benign disease of the uterus defined by the growth of the basal endometrium into the sub-endometrial myometrium (1). Due to postponing pregnancy until the third decade of a woman's life, it is much more recognized among infertile cases who underwent assisted reproductive technology (2). Adenomyosis manifests with dysmenorrhea, menorrhagia, pelvic pain, and an enlarged and tender uterus (3). The conceivable etiology is recommended as disturbance of the typical boundary between the endometrium and the myometrium during physiological recovery and mending of the endometrium, driving to myometrial intrusion by endometrium (4). Endometrial healing activates the immune system, resulting in increased estrogen following microtrauma to the endometrial-myometrial junction and increased uterine peristalsis activity (4, 5).

Adenomyosis's effects on fertility remains unclear, even though uterine peristalsis activity negatively affects sperm and embryo transfer (5). Based on the last meta-analysis published on the effect of adenomyosis on pregnancy outcomes following assisted reproductive technology, it is concluded that adenomyosis hurts in vitro fertilization clinical outcomes; therefore, pretreatment with the use of long-term gonadotropin-releasing hormone agonist (GnRHa) or long protocol could be advantageous (6).

Hormonal replacement therapy (HRT) is a common protocol for frozen embryo transfer (FET) cycles because of its more flexible program, enabling physicians to choose the day of embryo transfer suitable for women with or without regular ovulation. This protocol uses estrogen supplementation for endometrial preparation (7, 8). Pretreatment with a GnRHa added to the HRT protocol, results in better pituitary downregulation (9). The GnRHa protocol reduces tissue inflammation and angiogenesis and increases the apoptotic index, which improves endometrial receptivity in adenomyosis (5, 10).

Local hypoestrogenism is effective in myometrial hyperperistalsis activities and benefits implantation (11, 12). There is insufficient evidence to support preferring an endometrial preparation protocol with or without GnRHa for FET in these women. Li and colleagues, in a retrospective study, reported that pretreatment with GnRHa in an HRT cycle did not improve the clinical pregnancy or live birth rates of following FET among infertile women with adenomyosis (13).

Due to the deficiency of clinical trial studies on endometrial preparation using HRT cycles with and without GnRHa pretreatment in adenomyosis cases, we designed a randomized clinical trial to compare the pregnancy outcomes in infertile women with adenomyosis who underwent HRT cycles with and without long-term pituitary downregulation with GnRHa.

## 2. Materials and Methods

### Subjects

A phase III randomized clinical trial study was conducted on 140 adenomyosis cases, who underwent FET cycles at Arash Women's hospital (an academic hospital linked with Tehran Medical University), Tehran, Iran from May 2020 to March 2021.

The diagnostic criteria of adenomyosis in vaginal ultrasonography included: the presence of myometrial cysts, echogenic striation, nodules, cystic striation, myometrial thickening, enlarged global uterus, disrupted myometrial-endometrial junction, and posterior diameter of the myometrium that is larger than its anterior diameter (14, 15), which was accomplished by exclusively an expert gynecology sonographer using a PHILIPS EPIQ-7 3D ultrasonography machine (Netherlands). Adenomyosis was classified as diffuse and focal according to the ultrasonographic characteristics defined earlier (16).

The participants aged between 30-45 yr, who had a normal uterine cavity that underwent the 1 ${}^{\text{st}}$ or 2 ${}^{\text{nd}}$ cycle of FET and adenomyosis diagnosis that was either focal or generalized, with or without a concomitant diagnosis of endometriosis and myoma, were included in the study. The following diagnoses were considered as exclusion criteria: recurrent implantation failure, hydrosalpinx, uterine anomalies, severe male factor infertility, myoma > 5 cm, severe endometriosis, and uterine distortion due to myoma.

The history of menorrhagia, dysmenorrhea, or dyspareunia and other concomitant diseases, such as endometriosis and myoma diagnosis, have been examined and recorded.

### Sample size

The sample size was estimated to be a minimum of 130 (n = 65/ each) by considering the significance level of 5%, the power of 80%, according to the rate of clinical pregnancy between 2 groups (p1 = 51.35%) (p2 = 24.83%), and used the following formula based on the previous study (11). 


$$n=2\frac{{\left(Z_{1}-\frac{a}{2}+Z_{1}-\beta \right)}^{2}\bar{p}\bar{q}}{{\left(P_{1}-P_{2}\right)}^{2}}$$

### Randomization and blinding

Participants were allocated randomly into HRT and HRT+GnRHa pretreatment group (HRT+GnRHa) (n = 70/ each). The block randomization method was designed by the epidemiologist using STATA software, version 13. The type of study group was placed in a sealed envelope, and when the physicians approved the participants' eligibility for the study, the midwife then delivered the envelope to them. The researcher who followed up on the pregnancy results and the statistician did not know about the study grouping of the participants.

### Embryos assessment and endometrial preparation protocols

All top-quality embryos were frozen by the vitrification strategy 3 days after ovum pick-up for the research. Briefly, the embryo morphology was defined according to the criteria of previous study by evaluating the number and normality of blastomeres arrangement and the degree of fragmentation on the 3 ${}^{\text{rd}}$ day (17).

Due to the need for less hormonal monitoring, fewer women visit for ultrasound monitoring, and greater flexibility is observed in determining the day of embryo transfer according to the physician and individual preferences, HRT cycles were used instead of natural processes for FET embryo transfer.

In the HRT+GnRHa group, 2 doses of leuprolide acetate (Diphereline; Ipsen, France) 3.75 mg were prescribed through the early follicular stage of the menstrual cycle and 28 days afterward, and the HRT protocol was initiated in the following 10-14 days. In both groups, 2 mg estradiol valerate (Abureyhan, Iran) was administered daily, increasing the dose to 6 mg daily. A vaginal ultrasound was performed 10-14 days after estrogen supplementation to monitor endometrial thickness. When endometrial thickness exceeded ≥ 8 mm, progesterone supplement (an injectable progesterone 100 mg/daily) (Abureyhan, Iran) was administered, and frozen embryo replacement was planned.

The number of transferred embryos was chosen based on the maternal age and embryo quality. 2 or a maximum of 3 cleavage-stage embryos were for the most part transferred on the 4 ${}^{\text{th}}$ day of progesterone supplementation exclusively by an expert gynecologist using a cook embryo transfer catheter (MODEL: Arbor Medical Kororacija).

Embryo transfer was canceled if the optimal endometrial thickness was not achieved after 20 days. Serum β-human chorionic gonadotropin levels were measured 14-16 days after ET. If the test was positive, daily estradiol valerate (Abureyhan, Iran) and progesterone supplementation (Abureyhan, Iran, 50 mg/twice a day) were continued until the 12 ${}^{\text{th}}$ wk of pregnancy.

### Endometrial pattern evaluation

The endometrial pattern was determined 10-14 days after estradiol administration by transvaginal ultrasound, which was classified into 3 patterns; [Pattern A: Triple-line pattern consisting of the central hyperechoic line surrounded by 2 hypoechoic layers; pattern B: an intermediate isoechoic pattern with the same reflectivity as the surrounding myometrium; and pattern C: a poorly defined central echogenic line, resembling homogenous hyper echogenic endometrium] (18).

### Outcomes

The primary outcomes were chemical (positive beta human chorionic gonadotropin test 14 days after ET) and clinical pregnancy (the presence of at least 1 fetus with heart activity at 4-6 wk following ET) rates. Secondary outcomes, including twin pregnancy, miscarriage, and live birth rates, were followed up and reported. A twin pregnancy was detected on observation of 2 gestational sacs and the fetal poles with heartbeat in an ultrasound examination at 6-7 wk following ET. The miscarriage rate is considered as the loss of a clinical pregnancy before 20 wk of gestation. Live birth was defined as the delivery of a fetus that shows evidence of life after being entirely outside of the uterus, regardless of the duration of pregnancy. The pregnancy outcomes were reported per ET cycles, and twin births were considered as one live birth.

### Ethical considerations

The intention of the research was clarified to the samples, and all of them completed the informed agreement form, which was ready according to the Declaration of Helsinki. This study was approved by the Ethics Committee of Arash hospital of Tehran University of Medical Sciences, Tehran, Iran (Code: IR.TUMS.MEDICINE.REC.1399.034) and is registered on the IRCT website, which has been updated on January 01, 2023.

### Statistical analysis

Mean ± standard deviation (SD), frequency, and percentages were used to report quantitative and qualitative variables. For continuous variables, we first checked the normal distribution using the Kolmogorov-Smirnov test. To analyze normal distribution data, we used an independent sample *t* test, and if data were skewed Mann-Whitney U test was used. To analyze categorical variables, we used Chi-square or Fisher's exact test as needed. Univariable and multivariable logistic regression analysis were performed to detect significant related factors to clinical pregnancy rate individually and in a group model. All statistical analyses were performed using statistical package for the social sciences (SPSS, version 26, SPSS Inc., Chicago, IL), and statistical significance was assumed at p < 0.05.

## 3. Results

**Figure 1:**
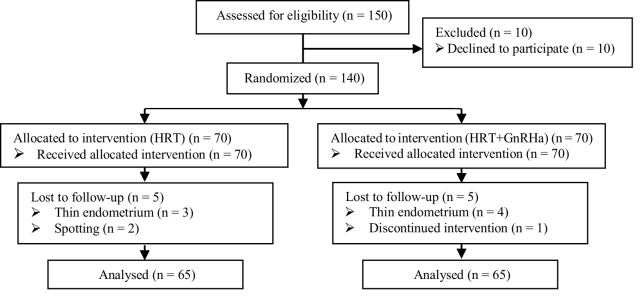
Consort flowchart of participants in the study.

Among 140 participants, 5 and 4 FET cycles were canceled in HRT with and without GnRHa pretreatment groups, respectively, and one woman avoided participating in the study plan. The final analysis included 130 cases with adenomyosis (Figure 1).

The demographic and baseline clinical characteristics of the cases were compared between groups. The means of women's age at transfer time had no significant difference between groups (p = 0.63). Also, no significant difference was observed between groups in terms of body mass index, type of infertility, cause of infertility, duration of infertility, and ET failure history. The women in pretreatment with the GnRHa group were more likely to have a previous history of FET cycles. In addition, the means of endometrial thickness at starting progesterone administration day were similar in both groups, and the predominant pattern in both groups was the triple line pattern (p = 0.56). No significant differences were observed between groups in the number of transferred embryos and embryo quality. Total estrogen dose and duration of its consumption were significantly higher in HRT with the GnRHa pretreatment group (p < 0.001, p < 0.001, respectively) (Table I).

In the follow-up, the chemical pregnancy, clinical pregnancy rates, as well as twin pregnancy, miscarriage, and live birth rates were found to be comparable between groups (p > 0.05) (Table II). All possible related factors were entered in the multivariable logistic regression model to independently identify the significant predictive factors to the clinical pregnancy rate. No significant variables were found (Table III).

**Table 1:** The comparison of demographic and baseline clinical characteristics between groups (n = 65/each)


**Variables**		**HRT**	**HRT+GnRHa**	**P-value**
Age at transfer (yr)*		35.81 ± 4.29	36.2 ± 4.79	0.63
Body mass index (kg/m^2^)*		24.98 ± 3.39	26.08 ± 3.56	0.07
Type of infertility**				
	**Primary**	47 (72.3)	42 (64.6)	
	**Secondary**	18 (27.7)	23 (35.4)	0.34
Infertility duration (yr)*		5.04 ± 1.95	5.06 ± 2.54		0.96
Type of adenomyosis**				
	**Diffuse**	58 (89.3)	60 (92.7)	
	**Focal**	7 (10.7)	5 (7.3)	0.54
Diagnosis of myoma**		4 (6.1)	5 (7.7)		0.73
Diagnosis of endometriosis (grade 1 and II)**		11 (16.9)	9 (13.8)	0.83
Cause of infertility**				
	**Female factor**	37 (56.9)	27 (41.5)	
	**Male factor**	15 (23.1)	15 (23.1)	
	**Mixed factor**	13 (20)	23 (35.4)	0.11
Number of the previous ET failure*		0.66 ± 0.43	0.61 ± 0.56		0.21
ET failure history**		19 (29.2)	28 (43.1)		0.14
<**Endometrial thickness at starting progesterone administration** day (cm)*		9.66 ± 1.3	9.79 ± 1.61	0.63
Endometrium pattern at starting progesterone administration day**				
	**Hyper echo**	0	1 (1.5)	
	**Intermediate isoecho**	4 (6.2)	5 (7.7)	
	**Triple line**	61 (93.8)	59 (90.8)	0.56
Number of transferred embryos*		2.53 ± 0.83	2.72 ± 0.64		0.16
Total estrogen days*		14.01 ± 2.05	15.2 ± 2.39		< 0.001
Total estrogen dose (mg)*		66.09 ± 12.34	73.66 ± 13.84	< 0.001
Embryo quality**				
	**Good**	60 (92.3)	58 (89.2)	
	**Fair**	5 (7.7)	7 (10.8)	0.54
*Data presented as Mean ± SD. Independent sample *t* test. **Data presented as n (%). Chi-square test. HRT: Hormone replacement therapy, GnRHa: Gonadotropin-releasing hormone agonist, ET: Embryo transfer				

**Table 2:** The comparison of treatment outcomes between groups (n = 65/each)


**Variables**	**HRT**	**HRT+GnRHa**	**P-value**
**Chemical pregnancy rate **	22 (33.8)	20 (30.8)	0.71
**Clinical pregnancy rate **	18 (27.7)	19 (29.2)	0.84
**Miscarriage rate **	5 (10.8)	5 (10.8)	-
**Twin pregnancy rate **	1 (1.5)	6 (9.2)	0.11
**Live birth rate per **	13 (20.1)	14 (21.5)	0.89
Data presented as n (%). Chi-square test. HRT: Hormone replacement therapy, GnRHa: Gonadotropin-releasing hormone agonist			

**Table 3:** Univariable and multivariable logistic regression analysis for detecting prognostic factors regarding clinical pregnancy rate


**Variables**		**Univariable logistic regression**			**Multivariable logistic regression**		
		**B (SE)**	**OR (95% CI)**	**P-value**	**B (SE)**	**OR (95% CI)**	**P-value**
**Woman's age (yr)**		-0.05 (0.04)	0.95 (0.86-1.04)	0.27	-0.15 (0.08)	0.85 (0.73-1)	0.06
**Woman' BMI (kg/m^2^)**		0.01 (0.05)	1.01 (0.91-1.13)	0.79	0.04 (0.06)	1.04 (0.93-1.17)	0.45
**Cause of infertility**							
	**Male factor**	0.64 (0.57)	1.90 (0.61-5.86)	0.26	1.11 (0.88)	3.03 (0.53-17.32)	0.21
	**Female factor**	-0.19 (0.52)	0.82 (0.29-2.29)	0.71	0.17 (0.72)	1.19 (0.28-4.94)	0.81
	**Mixed factor**	Reference	- -	- -	-
**Number of the previous ET**		1.20 (1.10)	3.32 (0.38-28.98)	0.27	1.96 (1.19)	7.15 (0.68-74.86)	0.99
<**No. of good quality** **transferred embryos**		0.42 (0.26)	1.58 (0.95-2.64)	0.07	0.50 (0.27)	1.66 (0.97-2.84)	0.06
**Total estrogen dose (mg)**		0.005 (0.014)	1.005 (0.97-1.03)	0.75	0.002 (0.016)	1.002 (0.97-1.03)	0.90
**Study groups**							
	**HRT**	0.001 (0.41)	1.0 (0.45-2.22)	0.99	0.64 (0.62)	1.89 (0.55-6.40)	0.30
	**HRT+GnRHa**	Reference	- -	- -	-
BMI: Body mass index, OR: Odds ratio, CI: Confidence interval, HRT: Hormone replacement therapy, GnRHa: Gonadotropin-releasing hormone agonist, ET: Embryo transfer							

## 4. Discussion

This clinical trial study demonstrated similar clinical and live birth rates in adenomyosis cases who underwent HRT with and without GnRHa pretreatment for FET cycles. Limited retrospective studies have compared the artificial FET cycle following GnRHa pretreatment to HRT, even though most of them suggest improvement of pregnancy outcomes in women with adenomyosis who underwent GnRHa pretreatment. In a retrospective study, it was described that on the day of progesterone administration, endometrial thickness was higher in HRT cycles; however, in the HRT group with pituitary downregulation by GnRHa, clinical pregnancy, implantation, and ongoing pregnancy rates were remarkably higher than that of HRT group, propounding improved pregnancy results in women with adenomyosis who experienced HRT FET cycle with GnRHa pretreatment (11).

The pregnancy outcomes have been compared after fresh ET cycles with and without GnRHa pretreatment and FET cycles following GnRHa pretreatment in a retrospective study. GnRHa pretreatment are associated with higher duration of the stimulation and total dose of gonadotropin, which led to a notably higher number of recovered oocytes in fresh cycles. The clinical pregnancy rate in group C (FET cycles) tended to be higher than those in groups B (fresh with) and A (fresh without), but without a significant difference (19). Recently, the pregnancy outcomes between women who underwent HRT cycles with and without GnRHa pretreatment have been compared in a large retrospective cohort study. They suggested GnRHa pretreatment may improve clinical pregnancy and live birth rate in women with endometriosis (20).

Adenomyosis is an estrogen-responsive disease, and pituitary downregulation with GnRHa for 1-3 months might increase the pregnancy rate (5). GnRHa decreases the inflammatory reaction and angiogenesis in adenomyosis tissues. Also, as GnRHa suppress the hypothalamus–pituitary–ovarian axis and induce a hypoestrogenic situation, leading to adenomyosis cell apoptosis and reducing the uterine size (4, 10, 21). Supraphysiologic estrogen concentrations lead to decreased integrin β3, osteopontin, and leukemia-inhibiting factor as endometrial receptivity markers during the endometrium implantation window in adenomyosis (2, 22).

In the current study, no superiority was observed between HRT+GnRHa and HRT cycles in pregnancy outcomes. On the other hand, we showed that the HRT cycle with GnRHa pretreatment causes significantly prolonged days and doses of estradiol consumption. In agreement with the present finding, in a retrospective study, it was concluded that GnRHa downregulation based on an HRT for the endometrial preparation in the FET cycle among infertile women with adenomyosis did not improve the rates of clinical pregnancy or live birth compared to HRT alone (13).

Due to the shorter time reaching embryo transfer in the HRT group and a lower rate of side effects and costs compared with GnRHa, this protocol is more patient-friendly than the HRT+GnRHa protocol, simultaneously the GnRHa could result in the delayed resumption of spontaneous ovulation after the FET cycle failures (23, 24).

The design of a clinical trial with a significant sample size is the strength of the present study. It was ideal to transfer a single euploid blastocyst to control the embryo quality factor; however, we could not apply this factor in our study due to limitations. Since the 2 groups were homogeneous in terms of baseline characteristics and no significant variable affecting clinical pregnancy was found in logistic regression tests, the findings of the present study are reliable.

## 5. Conclusion

The present study indicated that endometrial preparation for FET with and without suppression by GnRHa provides similar results in pregnancy outcomes. Moreover, the HRT cycle time interval to embryo transfer is shorter, so this protocol is much simpler, better, and more cost-effective than the HRT cycle with GnRHa pretreatment. Furthermore, prospective RCTs are needed to validate the optimal protocol for FET cycles in cases with adenomyosis.

##  Conflict of Interest 

The authors declare that there is no conflict of interest.
